# Cooperative Surveillance and Pursuit Using Unmanned Aerial Vehicles and Unattended Ground Sensors

**DOI:** 10.3390/s150101365

**Published:** 2015-01-13

**Authors:** Jonathan Las Fargeas, Pierre Kabamba, Anouck Girard

**Affiliations:** Department of Aerospace Engineering, University of Michigan, Ann Arbor, MI 48105, USA; E-Mails: daninman@umich.edu (P.K.); anouck@umich.edu (A.G.)

**Keywords:** sensor networks, target tracking, path planning for multiple UAVs

## Abstract

This paper considers the problem of path planning for a team of unmanned aerial vehicles performing surveillance near a friendly base. The unmanned aerial vehicles do not possess sensors with automated target recognition capability and, thus, rely on communicating with unattended ground sensors placed on roads to detect and image potential intruders. The problem is motivated by persistent intelligence, surveillance, reconnaissance and base defense missions. The problem is formulated and shown to be intractable. A heuristic algorithm to coordinate the unmanned aerial vehicles during surveillance and pursuit is presented. Revisit deadlines are used to schedule the vehicles' paths nominally. The algorithm uses detections from the sensors to predict intruders' locations and selects the vehicles' paths by minimizing a linear combination of missed deadlines and the probability of not intercepting intruders. An analysis of the algorithm's completeness and complexity is then provided. The effectiveness of the heuristic is illustrated through simulations in a variety of scenarios.

## 1. Introduction

A team of small unmanned aerial vehicles (UAVs) is tasked with patrolling a network of roads near a friendly base. Ground intruders (e.g., trucks) use the road network to reach the base and do not know of the presence of the UAVs. The UAVs patrol the roads to detect and take pictures of any intruders present on the roads.

However, small UAVs possess limited onboard processing resources, and the current detection capability of small aircraft using electro-optical or infrared sensors is not sufficient to ascertain whether an intruder is present or not [1]; thus, the UAVs in this problem are assumed to not possess automated target recognition capabilities. Instead, intruder detection is performed by unattended ground sensors (UGSs) placed on the roads. These sensors measure a given property (e.g., weight of a vehicle driving by), perform classification on the measurement to decide whether it corresponds to an intruder or not and register the time of the detection if the measurement was classified as an intruder. The use of UGSs in conjunction with UAVs enables the pursuit of an intruder along the road network, which is otherwise not possible solely using UAVs.

To maximize the coverage of the road network, the UGSs are placed far apart. The UGSs possess short-range communication devices, but have limited long-range communication capabilities; they require a line of sight to a dedicated communication device with a permanent power source. Placing communication devices to meet these requirements can be difficult depending on the terrain and conditions (e.g., contested environment). However, this problem can be circumvented by using mobile UAVs instead of immobile communication devices. The UAVs do not require the difficult placement of communication devices and power sources in the area, such that the line of sight is maintained between devices; instead, they act as mobile communication devices by querying UGSs directly below using a short-range communication device. Thus, it is assumed that the UGSs cannot communicate with one another, but only with UAVs directly overhead. In contrast, the UAVs are capable of communicating between one another and a central authority (e.g., the base) via a low bandwidth, long-range communication link. The low bandwidth link allows for the transmission of small amounts of information, such as UGS detections or waypoints, but prohibits the transmission of large datasets, such as images. The link is assumed to cover the entire operating area. The short-range and long-range communication devices are assumed to transmit in the electromagnetic spectrum, and as such, communication times are small compared to the other time constants involved in the problem.

This work is motivated by base defense scenarios within the Talisman Saber biennial U.S./Australian military exercise [2], where UAVs are tasked with obtaining intelligence (e.g., location and imagery) about intruders. In these base defense scenarios, the UAVs have limited onboard processing capabilities and, thus, cannot autonomously detect intruders; the UAVs thus rely on UGSs for intruder detection, pursuit and interception [3].

**Figure 1.**
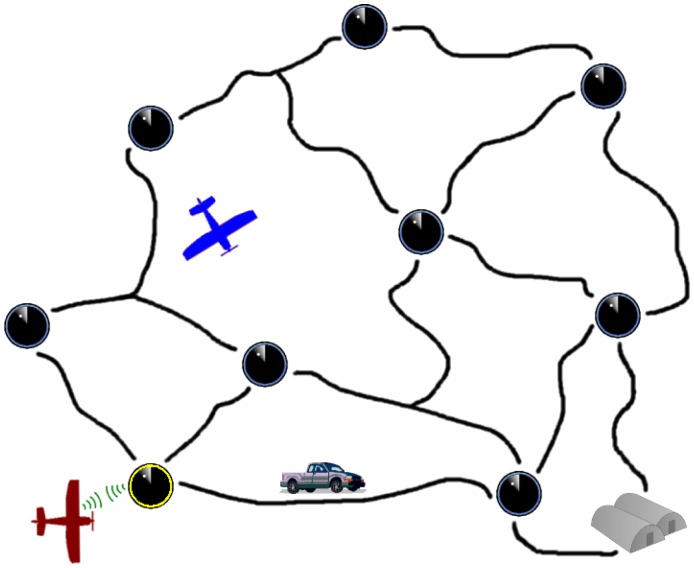
Cooperative surveillance and pursuit scenario.

Figure 1 shows a visualization of such a scenario with two UAVs and a single intruder attempting to reach the base. The UAV on the bottom of the figure is communicating with a UGS directly below. Through the communication, the UAV learns of a recent detection from the UGS. The detection is shared with the central authority and used to predict possible future locations of the intruder. The central authority then selects destinations for the UAVs where they are likely to image the intruder.

A mission designer is assumed to have set the UGSs locations and patrol parameters before the mission begins; optimal UGSs placement and patrol parameter selection for this base defense scenario is treated in [4].

The UAVs are forced to revisit UGSs to maintain up-to-date and accurate information on the intruder; this is enforced through the use of revisit deadlines, *i.e.*, the maximum time that can elapse between two consecutive visits to a UGS. Forced revisits also help mitigate the impact of false alarms by UGSs. The UAVs cannot detect a target autonomously; hence, they loiter above a UGS and capture an image when the UGS below detects an intruder passing.

The objective is to generate paths for the UAVs (*i.e.*, select waypoints in real time) that satisfy the revisit constraints of UGSs and capture images of intruders before the latter reach the base (*i.e.*, maximize the likelihood of a UAV and intruder being at the same location). A heuristic is provided to compute such paths, and its completeness and complexity are assessed. While the heuristic directs the paths taken by the UAVs, it also indirectly interacts with the UGSs used for monitoring the roads. The heuristic selects when information from the UGSs is obtained, and the heuristic uses the information acquired from the UGSs to predict possible intruder locations in the future. In addition, the capture of an image of the intruder is achieved when a UGS that a UAV is loitering (as directed by the heuristic) detects an intruder.

### 1.1. Literature Review

The UAVs' task consists of patrolling the road network and pursuing the intruders. A broad amount of research exists on patrolling problems and pursuit-evasion games; as such, literature pertaining to these topics is reviewed.

The problem of persistently monitoring a given area with one or more vehicles has been studied extensively. The problem of finding cycles, such that all points in the patrol area are covered by the vehicle's sensor footprint, is treated in [5]. In [6,7], the authors investigate a patrolling problem for multiple UAVs and maximize the minimum frequency of visitation for different partitions in the area. In [8,9], the authors find paths with a minimized uniform frequency of visitation for all partitions. In [10], the authors investigate the problem of finding paths for mobile agents that minimize the accumulation of an uncertainty metric in the mission area. In [11,12], the authors investigate heuristics, such that the time between visits to the same object of interest is minimized. The problem of patrolling multiple targets cooperatively is also treated in [13], where the authors provide algorithms to compute trajectories for vehicles that minimize the weighted refresh time of the targets being visited. The dual objective of satisfying patrolling constraints (revisit deadlines) and intercepting the intruder separates the problem studied in the current paper from many patrolling problems investigated in the literature.

This problem also shares similarities to the traveling salesman problem (TSP), where an agent is tasked with visiting a certain number of locations while minimizing the distance traveled. TSP is a non-deterministic polynomial-time (NP)-complete problem, and thus, heuristics are used to find solutions; [14] contains a survey on existing heuristics for TSP. In [15], the authors study stochastic and dynamic variations of TSP, where the target locations are generated randomly; the authors then provide algorithms to compute paths for the vehicles that optimize criteria, such as the length of the path or the time between the generation of the target and its observation by the vehicle. The problem treated in the current paper differs from vehicle routing problems and TSPs due to the added goal of capturing images of mobile intruders.

The problem considered in this paper has several components in common with pursuit-evasion games, where defenders are to capture an intruder. Pursuit-evasion games often occur on graphs; the defenders win the game if the intruder is caught; otherwise, the intruder wins. The defenders and intruder move in turns and can only travel to adjacent nodes. The pursuit-evasion problem was studied, and conditions on the number of defenders necessary to guarantee capture were derived in [16–18]. Many variations of the problem exist, such as the addition of constraints on the topology of the graph, the velocities of the defenders or intruders and the amount of information held by one team about the graph or the other team; a survey of pursuit-evasion research relevant to mobile robotics is presented in [19]. Security games introduce targets that the defenders must protect from intruder attacks; in addition, the intruder can observe the defenders. In [20,21], the authors study and provide algorithms to solve security games where the intruder is attempting to infiltrate nodes in a graph, and defenders must visit the various nodes in the graph, such that the intruder never has enough time to infiltrate. The current paper differs from the pursuit-evasion literature, because the defenders and intruders do not move in turns.

In [22], the problem of monitoring an area with intruders using UGSs and UAVs is discussed, and approaches to finding paths for the UAVs that maximize the number of interceptions are presented. The related problem of designing a sensor network for surveillance of a moving target has been studied in [23], where the authors provide methods for multi-point surveillance and demonstrate their effectiveness with regards to tracking probability. The problem of estimating a vehicle's position in a graph given its velocity distribution and a previous detection at a known location and time is treated in [24]. The authors use a histogram filter to predict the vehicle's potential locations in a graph. The approach used in this paper coordinates multiple UAVs (using a different method for prediction) to intercept an intruder on a graph in a centralized fashion while handling uncertainty in the target motion.

While the current literature examines many variations of patrolling problems and pursuit problems, no method treats a framework where the mobile agents fully rely on static sensors placed on an arbitrary road network to track and intercept intruders and provides a path planning algorithm for the mobile agents. The current paper concentrates on this subject. Addressing the reliance on static sensors is important, since the proper tracking of a target by a pursuer cannot be guaranteed in a large number of scenarios and, in some cases, may not be possible at all (as was indicated earlier with small UAVs currently being incapable of autonomous target recognition). In addition, not making assumptions about the topology of the network enables the handling of cases where the environment does not permit favorable sensor placement.

### 1.2. Original Contributions

The original contributions of this work are as follows: the problem of cooperative surveillance and pursuit is formulated and shown to be NP-hard; a heuristic to solve the problem is given; and an analysis of the heuristic's completeness and complexity is provided.

In previous work, the persistent visitation problem was introduced in which a single vehicle persistently visits a set of nodes, each with a revisit deadline [25]. The goal was to find a path for the vehicle such that no revisit deadline is missed. The existence of periodic solutions was proven; the complexity class of the problem was derived; and heuristics that could solve the problem were presented and characterized.

A version of this problem that included fuel constraints and refueling costs was also studied, and an algorithm that found the minimal cost path satisfying the revisit deadlines and fuel constraints was provided [26].

The current paper differs from the authors' previous work, which focused solely on patrolling, in the following ways: multiple vehicles are considered; an adversary is present in the area; sensors that provide information from which decisions need to be made are included; and the vehicles pursue the adversary while patrolling a number of locations with revisit deadlines.

### 1.3. Paper Outline

The remainder of the paper is as follows. In Section 2, the model for the defenders and intruders is presented, including the kinematics of the mobile agents and the properties of the UGS. The mathematics for the defenders' pursuit of the intruder are also presented in Section 2. The problem is formulated in Section 3. The heuristic that generates the defenders' actions to solve the problem is presented in Section 4. Results from simulations are shown in Section 5 and discussed in Section 6. Conclusions and future work are discussed in Section 7.

## 2. Modeling

In this section the model for the defenders (UAVs and UGSs) is presented, followed by a description of the intruder model.

### 2.1. Defenders

There are *n* UGSs placed in a planar area along a road network with Cartesian coordinates (*ξ_i_*, *ζ_i_*), 1 ≤ *i* ≤ *n*. The UGSs and the roads that the intruder can use to travel between them are modeled as a graph *G*(*N*, *E*), where *e*_*i*,*j*_ ∈ *E*, if there exists a road connecting UGSs *i* and *j*. Let *d*_*i*,*j*_ be the length of road *e*_*i*,*j*_. The road network and UGSs' placement is assumed to remain the same over the duration of the mission. The UGSs can measure a continuous property in their proximity and make a classification decision based on the measurement; this classification results in a detection if the measurement is above a certain threshold, e.g., in [22], the UGSs use a small Doppler radar to detect intruders nearby. The UGSs are not equipped with perfect sensors or classifiers and, thus, may emit false alarms.

In addition, *m* UAVs are patrolling the area each with constant velocity *v*, finite fuel capacity *F* and fuel consumption rate *ḟ_c_*. There is a single base that is capable of refueling the UAVs located at (*ξ_n_*_+1_, *ζ_n_*_+1_). The UGS are assumed to be distant from one another; thus, the UAVs' limited turn radius is not taken into account, and the UAVs are modeled as point masses moving in straight lines [27]. If straight line travel cannot be assumed, then curvature must be accounted for; this can be done using the results from [28], where the authors describe methods to convert paths on a graph with straight line edges to paths on a graph with edges that satisfy the turning constraints of the UAVs.

The UAVs are equipped with a long-range communication device, which enables communication with a central authority, and a short-range communication device, which enables the UAVs to query the status of a UGS directly below (in [22], short-range communication is performed via WiFi radios). The UAVs, UGSs and central authority are assumed to possess synchronized clocks; this can be achieved by calibration before the mission and periodic UGS clock synchronization during UAV visits (it can also be accomplished by equipping each UAV and each UGS with a clock synchronization device, such as a GPS).

Once a UAV obtains information from a UGS, it is immediately relayed to the central authority. The central authority decides which nodes the UAVs are to visit next once their respective destinations are reached. There is no benefit from allowing multiple UAVs to visit the same UGSs simultaneously, since a detection is shared immediately and only a single UAV is required to capture an image; hence, a UGS can only be visited by a single UAV at a time.

#### 2.1.1. Revisit Deadlines

Each UGS has a revisit deadline, *r_i_* > 0,1 ≤ *i* ≤ *n*, set by the mission designer, which under ideal conditions, is the maximum time that can elapse between two visits to the UGS by the defenders. The revisit deadlines are used as a method to keep the defenders' knowledge of intrusions up-to-date. The revisit deadlines are also used to indicate the relative importance of the various UGSs, such that the UAVs can prioritize their actions accordingly.

#### 2.1.2. State Space Model

UGS queries and intruder interceptions only occur above UGSs, and as such, the arrival of a UAV at a UGS is the event that advances the system for the defenders. Thus, a number of states evolve in discrete time corresponding to the arrival of a UAV at a UGS, while other states (such as the amount of fuel onboard a UAV) evolve in continuous time.

The increments (or steps) in discrete time are delimited by the arrivals of UAVs at UGSs. *τ*(*k*) ∈ ℝ. is the total time elapsed since the beginning of the mission upon completion of step *k*. Ω(*k*) is the set of upcoming UAV arrival times at step *k*. The current destination of UAV *j* at step *k* is indicated by *p_j_*(*k*) ∈ 1,2,…,(*n* + 1),1 ≤ *j* ≤ *m*. The Cartesian coordinates of UAV *j* at step *k* are (*ξ_j_*′ (*k*), *ζ_j_*′ (*k*)) ∊ ℝ^2^, 1 ≤ *j* ≤ *m* Let *y*(*k*) denote the discrete time states at step *k*, *i.e.*, *y*(*k*) = [*τ*(*k*) Ω(*k*) *p_j_*(*k*) (*ξ_j_*(*k*),*ζ_j_*(*k*))].

The continuous time states are linked to the discrete time states by applying impulses in their dynamics if certain conditions are met when a step is completed. The amount of fuel UAV *j* is carrying at time *t* is indicated by *f_j_*(*t*) ∈ ℝ, 1 ≤ *j* ≤ *m*. Let *x_i_*(*t*) ∈ ℝ, 1 ≤ *i* ≤ *n* be the slack time of UGS *i* at time *t*, which indicates how much time remains before a visit to UGS *i* is overdue. Let the input *u_j_*(*k*) be the destination of UAV *j* at step *k*.

#### 2.1.3. Initial Conditions

The initial time is set to zero. The initial set of arrival times is initialized to the empty set. Without loss of generality, the UAVs are assumed to start at the base with full fuel capacity, and the slack time for each UGS is initialized to its respective revisit deadline, *i.e.*,

(1) $$\begin{array}{ll} \tau \left(0\right) & =0,\\ \Omega \left(0\right) & =\emptyset ,\\ p_{1}\left(0\right) & =n+1,\\ \left({\xi_{1}}^{\prime }\left(0\right),{\zeta_{1}}^{\prime }\left(0\right)\right) & =\left(\xi_{n+1},\zeta_{n+1}\right),\\ p_{2}\left(0\right) & =n+1,\\ \left({\xi_{2}}^{\prime }\left(0\right),{\zeta_{2}}^{\prime }\left(0\right)\right) & =\left(\xi_{n+1},\zeta_{n+1}\right),\\ & \vdots \\ p_{m}\left(0\right) & =n+1,\\ \left({\xi_{m}}^{\prime }\left(0\right),{\zeta_{m}}^{\prime }\left(0\right)\right) & =\left(\xi_{n+1},\zeta_{n+1}\right),\\ f_{1}\left(0\right) & =F,\\ f_{2}\left(0\right) & =F,\\ & \vdots \\ f_{m}\left(0\right) & =F,\\ x_{1}\left(0\right) & =r_{1},\\ x_{2}\left(0\right) & =r_{2},\\ & \vdots \\ x_{n}\left(0\right) & =r_{n} \end{array}$$

#### 2.1.4. Dynamics

Let *q_j_*(*i*, *k*) be the Euclidean distance between UAV *j* and UGS *i* at step *k*. When a UAV arrives at its destination, its next destination is set by the input, and the travel time to that destination is added to the set of arrival times:

(2) $$\begin{array}{ll} \text{if}\;q_{j}\left(p_{j}\left(k\right),k\right)=0, & \{\begin{array}{ll} p_{j}\left(k+1\right) & :=u_{j}\left(k\right)\\ \Omega \left(k\right) & :=\left\{\frac{q_{j}\left(u_{j}\left(k\right),k\right)}{\upsilon }\right\}\cup \Omega \left(k\right) \end{array}\\ \text{else}, & \begin{array}{ll} p_{j}\left(k+1\right) & :=p_{j}\left(k\right) \end{array} \end{array}$$

The time of the next step is set by the minimum UAV arrival time in Ω(*k*); that time is then removed from Ω(*k*):

(3) τ(k+1):=minimum(Ω(k))

(4) Ω(k+1):=Ω(k)\{τ(k+1)}

The amount of fuel onboard a UAV decreases as dictated by the fuel consumption rate and is reset to full capacity whenever a visit to the base occurs:

(5) $$\begin{array}{cc} {\dot{f}}_{j}\left(t\right)= & -{\dot{f}}_{c}+\sum_{l=1}^{k-1}{\dot{f}}_{c}\times \left(1-\delta_{p_{j}(l)p_{j}(l+1)}\times \delta_{\left(n+1\right)p_{j}\left(l\right)}\right)\times H\left(t-\tau \left(l\right)\right)\times H\left(\tau \left(l+1\right)-t\right)\\ & +\sum_{l=1}^{k}\delta_{\left(n+1\right)p_{j}\left(l\right)}\times \left(F-f_{j}\left(\tau \left(l\right)\right)\right)\times \delta \left(t-\tau \left(l\right)\right),t\leq \tau \left(k\right),1\leq j\leq m \end{array}$$

where *δ_ij_* is the discrete Kronecker delta function, *H*(*t* − *τ*(*l*)) is the Heaviside step function, and *δ*(*t* − *τ*(*j*)) is the continuous Dirac delta function. The slack time of a given UGS decreases linearly in time and is reset to its revisit deadline whenever it is visited by a UAV:

(6) $$\begin{array}{cc} {\dot{x}}_{i}\left(t\right)= & -1+\sum_{l=1}^{k-1}\sum_{j=1}^{m}\left(1-\delta_{p_{j}\left(l\right)p_{j}\left(l+1\right)}\times \delta_{ip_{j}\left(l\right)}\right)\times H\left(t-\tau \left(l\right)\right)\times H\left(\tau \left(l+1\right)-t\right)\\ & +\sum_{l=1}^{k}\sum_{j=1}^{m}\delta_{ip_{j}\left(l\right)}\times \left(r_{i}-x_{i}\left(\tau \left(l\right)\right)\right)\times \delta \left(t-\tau \left(l\right)\right),t\leq \tau \left(k\right),1\leq j\leq m \end{array}$$

### 2.2. Intruder

A single intruder is traveling on the road network at a time; but there may be multiple intruders over the course of the mission. The intruders move inside their adversary's territory and suspect that they are under observation. Thus, the intruders move stochastically to reduce the predictability of their actions. However, the intruders do not know how they are being observed and cannot perceive the UAVs flying above [3]. If the intruders can detect the UAVs in the area, then a different intruder model is needed, e.g., using results from the pursuit-evasion literature or Stackelberg games.

The intruder moves from one node to the next in the graph according to a first order Markov process, *i.e.*, the next location that it visits only depends on its current location. The central authority is assumed to possess the Markov model for the intruder's movement; this model could have been obtained through intelligence or deduced from prior observation. The intruder enters the graph at a random node according to the initial distribution of the Markov model. Since the intruder's movement is memoryless, the UAVs and central authority only use the most recent intruder detection for their computations and do not keep track of the trail of UGSs detections left by the intruder.

The distance traveled by the intruder at time *t* is modeled as the process *X_t_*:

(7) $$\begin{array}{ll} X_{0} & =0,\\ X_{t}-X_{\rho } & =N\left(\mu_{\upsilon }\times \left(t-\rho \right),\sigma_{\upsilon }^{2}\times {\left(t-\rho \right)}^{2}\right),t>\rho >0 \end{array}$$

where *μ_v_* > 0, *σ_v_* > 0. The fuel consumption of the intruder's vehicle is assumed to be negligible.

### 2.3. Intruder Interception

To direct the UAVs, such that interception is likely to occur, the probability of the intruder passing by a UGS that a UAV is loitering above must be calculated.

#### 2.3.1. Probability of Intruder Passing a Node in a Given Time Window

Given a path *s* of UGSs, where *s*(*i*) indicates the *i*—*th* UGS visited and the fact that the intruder passed *s*(1) at time zero, the probability of the intruder passing *s*(2) between times *t_i_* and *t_f_* (where *t_i_* > 0 and *t_f_* > *t_i_*) is:

(8) $$P_{\text{int}}\left(s\left(2\right),s,t_{i},t_{f}\right)=\int_{t_{i}}^{t_{f}}P\left[X_{\rho }=d_{s\left(1\right),s\left(2\right)}\right]d\rho$$

The probability of the intruder passing *s*(3) between times *t_i_* and *t_f_* is:

(9) $$P_{\text{int}}\left(s\left(3\right),s,t_{i},t_{f}\right)=\int_{t_{i}}^{t_{f}}P\left[X_{\rho }=d_{s\left(1\right),s\left(2\right)}+d_{s\left(2\right),s\left(3\right)}\right]d\rho$$

The probability of the intruder passing *s*(*b* + 1) between times *t_i_* and *t_f_* is:

(10) $$P_{\text{int}}\left(s\left(b+1\right),s,t_{i},t_{f}\right)=\int_{t_{i}}^{t_{f}}P\left[X_{\rho }=\sum_{f=1}^{b}d_{s\left(f\right),s\left(f+1\right)}\right]d\rho$$

Generalizing, the probability of the intruder passing node *j* (while traveling along the path *s*) between times *t_i_* and *t_f_* is:

(11) $$P_{\text{int}}\left(j,s,t_{i},t_{f}\right)=\sum_{b=1}^{\left|s\right|-1}\delta_{s\left(b+1\right),j}\int_{t_{i}}^{t_{f}}P\left[X_{\rho }=\sum_{f=1}^{b}d_{s\left(f\right),s\left(f+1\right)}\right]d\rho$$

The probability that the intruder will pass node *j* between times *t_i_* and *t_f_* before reaching the base is:

(12) $$P_{\text{int}}\left(j,\text{s},t_{i},t_{f}\right)=\sum_{b=1}^{\left|s\right|-1}\left(1-1_{s\left(1:b\right)}\left(n+1\right)\right)\delta_{s\left(b+1\right),j}\int_{t_{i}}^{t_{f}}P\left[X_{\rho }=\sum_{f=1}^{b}d_{s\left(f\right),s\left(f+1\right)}\right]d\rho$$

where **1***_s_*_(1_*_:b_*_)_(*n* + 1) is the indicator function.

A set of paths *S* is introduced, where *S*(*a*) indicates the ath path within the set and *S*(*a, f*) indicates the *f*th node visited in the *a*th path within the set. *P*(*S*(*a*)) is the probability of the *a*th path occurring in the set where $\sum_{a=1}^{\left|S\right|}P(S(a))=1;P(S(a))$ is computed using the Markov model for the intruder's motion along the road network. Thus, the probability that the intruder will pass node *j* between times *t_i_* and *t_f_* before reaching the base given a set of paths *S* is:

(13) $$P_{\text{int}}\left(j,\text{S},t_{i},t_{f}\right)=\sum_{a=1}^{\left|S\right|}P\left(S\left(a\right)\right)\sum_{b=1}^{\left|S\left(a\right)\right|-1}\left(1-1_{S\left(a,1:b\right)}\left(n+1\right)\right)\delta_{S\left(a,b+1\right),j}\int_{t_{i}}^{t_{f}}P\left[X_{\rho }=\sum_{f=1}^{b}d_{S\left(a,f\right),S\left(a,f+1\right)}\right]d\rho$$

#### 2.3.2. Probability of Intruder Interception

Let *g_i_*(*j*, *k*) be the Euclidean distance between the positions of UAV *i* at steps *j* and *k*. Given the probability of an intruder passing a UGS during a certain time window, the probability of intruder interception can be calculated by accounting for the UAV locations:

(14) $$\begin{array}{ll} P_{\text{capture}}\left(S,y\left(l\right),y\left(l+1\right)\right) & =\sum_{\text{j}=1}^{m}\alpha_{j}\times p_{\text{int}}\left(p_{j}\left(l+1\right),S,\tau \left(l\right),\tau \left(l+1\right)\right)\\ \text{where}:\alpha_{j} & =\{\begin{array}{cc} 1 & \text{if}\;{\text{g}}_{j}\left(l,l+1\right)=0\\ 0 & \text{otherwise} \end{array}. \end{array}$$

This equation consists of a sum over all of the UAVs, where *j* indicates the UAV index. *α_j_* is only one when UAV *j* is loitering over a UGS during the step, in which case, the probability of an intruder passing the node where the UAV is located during the time window of the step is added.

## 3. Problem Formulation

Using the models for the defenders and intruders presented in the previous section, the problem is now formulated. Given the UAV states' at the current step, the UGSs' revisit deadlines and the results from the UGSs' queries, the UAVs are to find paths that satisfy the revisit deadlines and maximize the probability of intercepting the intruder. This revisit deadline satisfaction version of the problem does not allow for missing any revisit deadline and, hence, limits the UAVs' ability to pursue the intruder.

Thus, a revisit deadline optimization version problem is formulated to give the UAVs flexibility in meeting revisit deadlines and pursuing the intruder. Instead of satisfying the revisit deadlines, the amount by which revisit deadlines are missed is minimized. Let *t_mission_* be the duration of the UAVs' base defense mission. Let *S_l_* be the set of possible intruder paths given the most recent intruder detection at step *l*. Let ⌊*h*⌋ (*u, t*) indicate the smallest step in sequence *u* of UAV actions where *τ*⌊*h*⌋ (*u*, *t*)) ≥ *t*. Let *x̃_i_*(*t*) and *x̰_i_*(*t*), respectively, be the slack time left and the slack time overdue at time *t*,

(15) $${\tilde{x}}_{i}\left(t\right)=\frac{\left(\left|x_{i}\left(t\right)\right|+x_{i}\left(t\right)\right)}{2}$$

(16) $${\underset{∼}{x}}_{i}\left(t\right)=\frac{\left(\left|x_{i}\left(t\right)\right|-x_{i}\left(t\right)\right)}{2}$$

Let *β* be the cost of not capturing the intruder, and let *γ* be the cost associated with missing a deadline, where *β* > 0 and *γ* > 0. Let *C*(*l*) be the cost of the UAV actions taken at step *l*,

(17) $$\begin{array}{cc} C\left(l\right) & =\beta \times \left(\tau \left(l+1\right)-\tau \left(l\right)\right)\times \left(1-P_{\text{capture}}(S_{l},y\left(l\right),y(l+1)\right)\\ & +\frac{\gamma }{n}\times \sum_{i=1}^{n}\left(\tau \left(l+1\right)-\tau \left(l\right)-{\tilde{x}}_{i}\left(\tau \left(l\right)\right)\right)\times \frac{{\underset{∼}{x}}_{i}\left(\tau \left(l+1\right)\right)+{\underset{∼}{x}}_{i}\left(\tau \left(l\right)\right)}{2} \end{array}$$

The first component of the cost penalizes the UAVs for not intercepting the intruder over the course of the step. The second component of the cost penalizes revisit deadlines that are overdue by adding the integral of the slack time missed over the course of the step.

Based on the cost function above, the revisit deadline optimization version of the problem is formulated as follows: the central authority is to find a sequence ***u****_j_*(***k***), **1** ≤ ***j*** ≤ ***m***, ***k* ∈** ℕ, such that, under Equations (1), (2), (3), (4), (5) and (6), $\sum_{k=1}^{\left\lfloor h\right\rfloor \left(u,t_{\text{mission}}\right)}C(k)$ is minimized and **0** ≤ ***t*** ≤ ***t****_mission_*, **1** ≤ ***j*** ≤ ***m***, ***f****_j_*(***t***) ≥ **0**.

### 3.1. Problem Complexity

#### Proposition 1

*The revisit deadline satisfaction version problem of cooperative surveillance and pursuit is NP-hard*.

#### Proof

If no intruders are present in the road network, then an optimal solution is one where the slack times are kept positive. The problem of keeping slack times positive for a single UAV is the persistent visitation problem and is proven to be NP-complete in [25]. The persistent visitation problem can be reduced to the problem of cooperative surveillance and pursuit by selecting one UAV to patrol the same graph with the same revisit deadlines without intruders present. Thus, the problem of cooperative surveillance and pursuit is NP-hard.

#### Corollary 2

*The revisit deadline optimization version problem of cooperative surveillance and pursuit is NP-hard*.

## 4. UAV Path Selection

The problem is NP-hard, hence a heuristic algorithm is used to select the paths of the UAVs. This algorithm searches ahead for a sequence of UAV actions that minimizes the cost function within a certain time window and executes the first set of UAV actions in the sequence.

### 4.1. System Structure

The algorithm used to simulate the defenders' system for a cooperative surveillance and pursuit problem is provided in Algorithm 1. The states are first initialized (Lines 1–3); then at each step, the possible intruder paths within *t_search_* are computed (Line 5), followed by the computation of the minimal cost action for the UAVs within the same time horizon using the possible intruder paths (Line 6). In addition, at each step *k*, new detections are added from the UGS queries (Lines 9–11), stale queries rejected (Lines 12–16) and UGS queries without detections added (Lines 17–18). A UGS query is characterized by the status of the UGS, the time of the detection if one occurred (otherwise, the time of the query) and the index of the queried UGS. The resulting data from querying the UGSs is used in the intruder path generation process in the next time step. This process of finding intruder paths, selecting UAV actions and gathering UGSs queries is repeated until the mission completion time is reached (Line 4).


**Algorithm 1:** Defenders' system for the cooperative surveillance and pursuit problem.
**Data:**
*G*(*N*, *E*), *r*,*d*,*m*,*v*,*F*,*ḟ_c_*,*t_mission_*,*t_search_*,*t_stale_*1*k* ← 0; *t* ← 02(*y*(*k*), *f*(*t*), *x*(*t*)) ← (Equation (1))3*t_D_* ← ∅; *n_D_* ← ∅; *T_U_* ← ∅; *N_U_* ← ∅4**while**
*τ(k*) < *t_mission_*
**do**5 (*S_k_*, *P*(*S_k_*)) ← *paths*(*n_D_*, 1, *t_D_*, *t_D_*, *T_U_, N_U_, τ*(*k*) + *t_search_*, ∅, ∅)6 *u*(*k*) ← *uavsAction*(*y*(*k*), *f*(·), *x*(·), *t_search_*, *S_k_, P*(*S_k_*))7  (*y*(*k* + 1), *f*(·),*x*(·))← (Equations (2), (3), (4), (5) and (6)), *y*(*k*), *u*(*k*)8 *k* ← *k* + 1; *t* ← *τ*(*k* + 1)9 **for**
*(detection)* ∈ *queries(k)*
**do**10  **if** (*detection*).*t* > *t_D_*
**then**11    (*t_D_*, *n_D_*) ← (*detection*)12 **if**
*t_D_* + *t_stale_* < *τ*(*k*) **then**13  *t_D_* ← ∅; *n_D_* ← ∅; *T_U_* ← ∅; *N_U_* ← ∅14  **for** (*t_U_*, *n_U_*) ∈ (*T_U_*, *N_U_*) **do**15   **if**
*t_U_* ≤ (*τ*(*k*) –*t_stale_*) **then**16    (*T_U_*, *N_U_*) ← (*T_U_*, *N_U_*) \ (*t_U_*, *n_U_*)17 **for**
*(*¬*detection)* ∈ *queries(k)*
**do**18  (*T_U_*, *N_U_*) ← (*T_U_*, *N_U_*) ← (¬*detection*)**Result**: *u_j_*(·)


### 4.2. Intruder Path Generation

Possible intruder paths are generated using the information from UGS queries. The central authority stores the time and UGS index of the most recent intruder detection. It also stores the times and UGS indices of recent UGS queries without detections. This information is used to generate the potential intruder paths at each step using a recursive breadth first search methodology. If a detection is too stale, then it is ignored. In simulations, the threshold for a detection to be considered stale was selected to be half the mean intruder travel time between the two UGSs most distant from one another.

A recursive algorithm is used to compute the possible intruder paths (Algorithm 2); this algorithm is used by the heuristic to assist in its decision making process. This algorithm to find intruder paths is the method by which the heuristic uses the information obtained from the UGSs. The input of the algorithm is the current candidate path for the intruder (which at the start of the algorithm's execution, is the most recent detection). At each iteration in the search, the nodes adjacent to the last node of the current working path, *s*, are obtained (Line 1). Possible travel times to these adjacent nodes are then computed using the intruder's velocity probability distribution. If any of the visits to the adjacent nodes violate the constraints set by recent UGS queries without detections, then they are removed (Lines 2–10). Valid adjacent nodes are then appended to the current working path (Lines 11–12). If the minimum travel time of the new path is larger than the search depth, then the path is admitted to the set of possible paths (Lines 14–15); otherwise, the search continues for that path (Lines 16–17). When all of the candidate paths reach the search depth, the algorithm terminates and returns the possible intruder paths. These possible intruder paths are then used to select the actions of the UAVs.


**Algorithm 2:** The paths()algorithm to find possible intruder paths.
**Data**: *s*,*p*,*t_min_*,*t_max_*,*T_U_*,*N_U_*,*t_f_*,*S*,*P*(*S*)1Γ ← *adjacentNodes*(*s*(|*s*|), *G*)); *l* ← |Γ|2**while**
*l* > 0 **do**3 
${T^{\prime }}_{\max}\left(l\right)\leftarrow t_{\max}+\frac{d_{s\left(\left|s\right|\right),\Gamma \left(l\right)}}{\min\left(\upsilon_{\text{int}}\right)}$4 **for** (*t_U_*, *n_U_*) ∈ (*T_U_*, *N_U_*) **do**5  **if** Γ(*l*) = *n_U_*
**then**6   **if**
${T^{\prime }}_{\max}\left(l\right)\leq t_{U}$**then**7    Γ ← Γ \ {Γ(*l*)}8    
${T^{\prime }}_{\max}\leftarrow {T^{\prime }}_{\max}\backslash \left\{{T^{\prime }}_{\max}\left(l\right)\right\}$9   **break**10 *l* ← (*l* — 1)11**for**
*l* ← 1 **to** |Γ| **do**12 
$s^{\prime }\leftarrow \left[s\Gamma \left(l\right)\right];p^{\prime }\leftarrow p\times \frac{1}{\left|\Gamma \right|}$13 
${t^{\prime }}_{\min}\leftarrow t_{\min}+\frac{d_{s\left(\left|s\right|\right),\Gamma \left(l\right)}}{\max\left(\upsilon_{\text{int}}\right)}$14 **if**
${t^{\prime }}_{\min}\geq t_{f}$**then**15  *S* ← *S* ∪ {*s′*}; *P*(*S* = *s′*) ← *p′*16 **else**17  (*S*,*P*(*S*)) ← *paths*
$\left(s^{\prime },p^{\prime },{t^{\prime }}_{\min},{T^{\prime }}_{\max}\left(l\right),T_{U},N_{U},t_{f},S,P\left(S\right)\right)$**Result**: *S*,*P*(*S*)


### 4.3. Selection of UAV Actions

The following algorithm is used by the heuristic to make its decisions. The algorithm starts by searching for all possible sequences of actions for the team of UAVs within the search horizon *t_search_*. For each sequence of actions available to the team of UAVs, *ũ*, it then assesses the cost using the following equation:

(18) $$\sum_{l=k}^{\left\lfloor h\right\rfloor \left(\tilde{u},t_{\text{search}}\right)}C\left(l\right)$$

where *k* is the current step. The cost calculations use the intruder paths generated earlier.

The algorithm starts by using the current state of the UAVs to compute feasible actions. The action is then applied, and the corresponding UAV states and costs are computed. These actions, states and costs are then added to sets of candidate sequences of actions, candidate states of the UAVs after the application of the corresponding sequence of actions and candidate costs after the application of the corresponding sequence of actions. The algorithm then proceeds to iterate over the set of candidate sequences of actions for the UAVs.

The procedure for computing potential sequences of actions and their costs is described in Algorithm 3. Several intermediate variables are used: *Ũ* is the working set of sequences of UAV actions; *W* is the corresponding set of states after the sequences of actions in *Ũ* have been applied; and Θ is the set of costs for the sequences of actions. *u* is the current minimal cost sequence of actions, and *φ* is the current minimal cost. These variables are initialized (Lines 1–2). The algorithm then proceeds to loop over the working set of sequences of UAV actions until none remain (Line 3). At each iteration, the first elements in the working sets of states, sequences of actions and costs are obtained and removed from their parent sets (Lines 4–5). The set of possible actions, *Z*, given the current working state *w* ∈ *W* is then computed (Lines 6–14). For each possible action for the team of UAVs, *z* ∈ *Z*, the next state, *λ*, is computed (Line 15). If *λ* results in positive fuel for all of the UAVs and has reached the search depth, then its cost is computed (Lines 16–17); if that cost is smaller than the current minimal cost, then the current minimal cost sequence of actions is set to the sequence of actions that resulted in *λ* (Lines 18–19). If *λ* results in positive fuel for all of the UAVs without reaching the search depth, then the working state, sequence of actions and cost are added to the corresponding parent working sets (Lines 20–24). The algorithm terminates when the set of candidate sequences of UAV actions is empty (*i.e.*, all feasible actions in the search horizon have been considered) and returns the minimal cost sequence of actions. The heuristic directs the UAVs to take the first step in this minimal cost sequence of actions; this step results in the UAVs visiting or loitering above certain UGSs, thereby obtaining new information from the UGSs and possibly capturing an image of the intruder. The possibility of imaging the intruder exists when a UAV is loitering above a UGS; the capture of an image is triggered when the UGS which the UAV is loitering detects the intruder.

### 4.4. Algorithm Completeness

The search depth for the UAV actions affects the existence of solutions; if the search depth is less than the endurance of the UAVs, *t_search_* < (*F*/*ḟ_c_*), then there is no guarantee that the generated paths will lead to the satisfaction of the UAVs' fuel constraints. If *t_search_* ≥ *t_mission_*, then the heuristic is complete, *i.e.*, it will find a solution if one exists.


**Algorithm 3:** The uavsAction() heuristic to find the minimal cost action for the UAVs within a given search horizon.
**Data**: *y*(*k*),*f*(·),*x*(·),*S*,*P*(*S*)1*W* ← {(*y*(*k*), *f*(·), *x*(·))}2*Ũ* ← ∅; Θ ← ∅;*u* ← ∅; *ϕ* ← ∞3**while** |*W*| > 0 **do**4 *w* ← *W*(1); *ũ* ← *Ũ* (1); *θ* ← Θ(1)5 *W* ← *W* \ {*w*}; *Ũ*← *Ũ* \ {*ũ*}; Θ ← Θ \ {*θ*}6 *Z* ← ∅7 **if**
*q*_1_(*w.p*_1_) = 0 **then**8  *Z* ← *N* ∪ {*n* + 1}9 **for**
*l* ← 2 **to**
*m*
**do**10  **if**
*q_l_*(*w.p_l_*) = 0 **then**11   *Z* ← *Z* × {*N* ∪ {*n* + 1}}12  **else**13   *Z* ← *Z* × ∅14 **for**
*z* ∈ *Z*
**do**15  *λ* ← (Equations (2), (3), (4), (5) and (6)), *w*, *z*16  **if** (∀*j*, *λ.f_j_*(*λ. τ*) ≥ 0) Λ (*λ*.*τ* ≥ *τ*(*k*) + *t_search_*) **then**17   *θ*′← *θ* + (Equation (17)), *S*, *P*(*S*), *w*, *λ*18   **if**
*θ*′ < *φ*
**then**19    *u* ← [*ũ z*]; *ϕ* ← *θ′*20  **else if** ∀*j*, *λ*.*f_j_*(*κ.τ*) ≥ 0 **then**21   *θ′* ← *θ* + (Equation (17)), *S*, *P*(*S*), *w*, *λ*22   **if**
*θ′* < *ϕ*
**then**23    *W* ← *W* ∪ {*λ*}; *Ũ* ← *Ũ* ∪ [*ũ z*]24    Θ ← Θ ∪ {*θ*′}**Result**: *u*


### 4.5. Algorithm Complexity

The topology of the road network, the number of UAVs, the UAVs' velocity, the mean intruder velocity and *t_search_* affect the algorithm complexity Let *d̄* be the mean distance between any two UGSs. By inspection, the time complexity of the heuristic per step is:

(19) $$O\left({\left|E\right|}^{\frac{t_{\text{search}}\times \mu_{\upsilon }}{\bar{d}}}+{\left(\frac{n!}{m!\left(n-m\right)!}\right)}^{\frac{t_{\text{search}}\times \upsilon }{d}}\right)$$

The complexity increases polynomially with respect to the number of UGS and the number of roads, increases exponentially with respect to the vehicle velocities and the search depth and decreases exponentially with respect to the mean distance between any two UGSs.

## 5. Simulations

To illustrate the performance of the *uavsAction*() heuristic, several simulations with varying configurations are shown. A local search heuristic is used as a baseline comparison to the *uavsAction*() heuristic developed in the previous sections. The local search heuristic is detailed in Appendix A.

**Figure 2.**
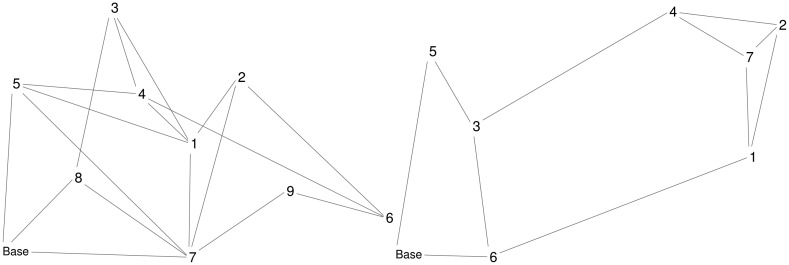
Visualization with base, UGSs and roads of Scenario A (**left**) and B (**right**).

Four scenarios are considered: Scenario A2 occurs in Area A with a UAV-to-intruder velocity ratio of two; Scenario A3 occurs in Area A with a UAV-to-intruder velocity ratio of three; Scenario B1 occurs in Area B with a UAV-to-intruder velocity ratio of one; and Scenario B2 occurs in Area B with a UAV-to-intruder velocity ratio of two. Two UAVs are operating in Area A, while three UAVs are operating in Area B; UAV fuel consumption is not accounted for in these simulations. Visualizations of Areas A and B are shown in Figure 2. Detailed parameters for the scenarios are given in Tables 1 and 2; these tables contain the location of the base and UGSs, revisit deadlines for the UGSs, and velocity for the UAVs and intruders.

**Figure 3.**
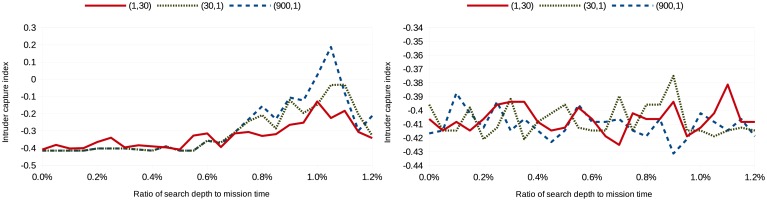
Intruder capture index for Scenario A2 using *uavsAction*() (**left**) and *localSearch*() (**right**).

**Figure 4.**
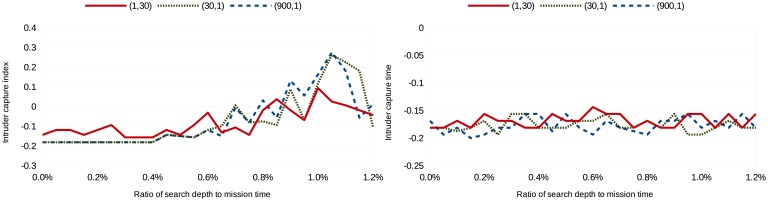
Intruder capture index for Scenario A3 using *uavsAction*() (**left**) and *localSearch*() (**right**).

**Figure 5.**
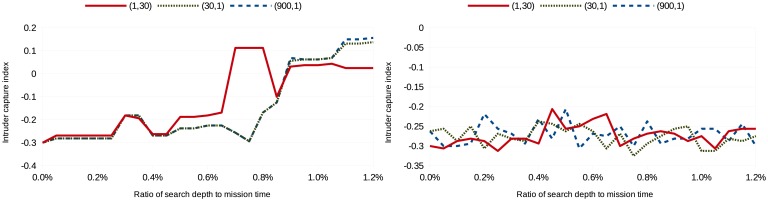
Intruder capture index for Scenario B1 using *uavsAction*() (**left**) and *localSearch*() (**right**).

**Figure 6.**
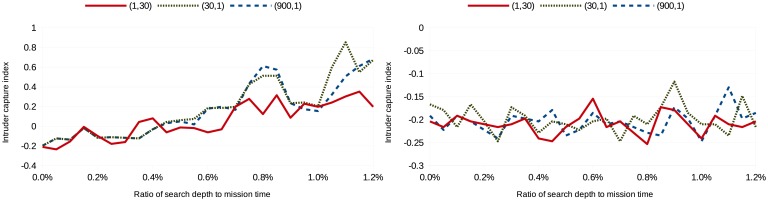
Intruder capture index for Scenario B2 using *uavsAction*() (**left**) and *localSearch*() (**right**).

The metric used to assess the performance of the approaches is the intruder capture index. The intruder capture index is the number of intruders whose image was captured subtracted by the number of intruders that reached the base divided by the total number of intruders over the course of the mission. Three cost configurations, indicated by (*β,γ*), are considered: (1, *t_mission_*), (*t_mission_*,1), $\left(t_{\text{mission}}^{2},1\right)$, where the mission time, *t_mission_*, is 30 in the simulations. Missing revisit deadlines is weighted heavily in the first cost configuration, while not capturing an image of the intruder is weighted heavily in the last cost configuration. Eighty simulations were run per search depth per scenario per cost configuration; Figures 3, 4, 5 and 6 show the average intruder capture index (represented on the ordinate) for these 80 simulations for the three different cost configurations (represented by the three different line styles) as a function of search depth for Scenarios A and B (represented on the abscissa).

In the left subfigures of Figures 3, 4, 5 and 6, where *uavsAction*() is used, the intruder capture index increases as a function of search depth for all cost functions. The intruder capture index for *localSearch*() (right subfigures of Figures 3, 4, 5 and 6) does not increase significantly with a larger search depth. While *uavsAction*() and *localSearch*() perform similarly for short search depths, *uavsAction*() performs better than *localSearch*() for larger search depths.

For Scenarios A2, A3 and B2 (Figures 3, 4 and 6), weighting intruder capture heavily when using *uavsAction*() resulted in more captures for large search depths, while only a marginal difference between cost configurations is seen for small search depths. In Scenario B1 (Figure 5), weighting missed revisit deadlines more heavily for *uavsAction*() resulted in more captures for search depths less than 0.9% of the mission completion time. The performance of *localSearch*() is not significantly affected by different cost configurations.

In Scenario A, an optimal search depth is seen for both velocity advantages at 1.05% of the mission completion time for *uavsAction*(). An optimal search depth is not seen in Scenario B; however, it may occur at a search depth greater than performed in the simulations. As expected, the intruder capture indices in Scenario A3 are larger than in Scenario A2, *i.e.*, the UAVs perform better when they are faster. This trait is also seen when comparing Scenario B2 to Scenario B1. The UAVs do not perform well when they do not have a velocity advantage (Figure 5), even when they are more numerous. When the UAVs are faster than the intruder and the topography is advantageous, as is the case in Scenario B, they can perform very well (Figure 6).

Videos of simulations for a variety of other scenarios can be found at [29].

## 6. Discussion

The performance of the *uavsAction*() heuristic largely depends on the problem instance, as can be seen from the differences in the results from the scenarios presented. In the simulations, the UAVs perform better with a larger velocity advantage compared to the intruder; this behavior is expected in all scenarios. Problem instances with bottlenecks in the road network topology lead to better performance, since the UAVs' pursuit and interception of the intruder are simplified. Topologies with highly connected nodes close together lead to poorer performance, since the UAVs cannot adequately pursue the intruder. The effect of the topology on the performance of the heuristic highlights the importance of the selection of UGSs' locations.

The simulations demonstrate that the heuristic can be used to plan the paths of UAVs in cooperative surveillance and pursuit tasks. Cooperative surveillance and pursuit systems, such as the motivating Talisman Saber exercise, are realistic and can be constructed with current technology. Details on a UAV and UGS system to monitor and pursue intruders for the Talisman Saber exercise is provided in [22]. This system uses small, commercially available UAVs and small Doppler radars as UGSs. UAVs and UGSs communicate using WiFi radios and the UAVs capture images of the intruder using a gimballed video camera. The Talisman Saber area is approximately 2450 km^2^, and the road network within the area is sparse. The velocity of the UAVs ranges between 50 km/h and 100 km/h, and typical cruising altitude ranges between 60 m and 900 m, which is within the 1-km communication range of the UGSs' WiFi radios. With these parameters, the simulations shown in Section 5 can be viewed as taking place in an area nine-times larger than the Talisman Saber area or with UAVs that travel at a third of the velocity of the commercially available UAVs discussed. Simulating faster UAVs in a smaller area with more UGSs, similar to Talisman Saber, takes much longer to simulate, because of the complexity of the heuristic: in Equation (19), faster UAVs and smaller areas increase the computation time exponentially, and increasing the network size increases the computation time polynomially.

While this work is motivated by the Talisman Saber exercise, the heuristic can be used for other monitoring and pursuit tasks occurring on graphs using vehicles and stationary sensors with the same assumptions and dynamics that are described above; examples of such tasks include a pollutant monitoring task in a body of water using autonomous boats in conjunction with stationary buoys or the monitoring of a forest fire or flood using appropriate sensors on the ground and autonomous aircraft to visit these sensors.

The assumptions made in this work allow for paths to be computed while still capturing the essence of the problem; however, they do not allow for the modeling of certain features that do occur in real scenarios. For example, the UAV constant velocity and constant fuel consumption rate assumption does not handle wind disturbances. Wind disturbances affect the speed of the UAVs and alter the arrival times of UAVs at UGSs. If the wind is steady, the decision making heuristic can account for wind in its computations. If the wind is stochastic, *i.e.*, turbulence, the constant fuel consumption rate assumption needs to be relaxed, so that more fuel can be consumed during turbulence to allow the vehicles to remain on their nominal paths. The assumption that the intruder moves according to a Markov chain may be valid in some scenarios, but does not allow for intelligent intruder behavior, such as reacting to seeing a UAV flying overhead or attempting to deceive the UAVs. The assumptions made for communication reflect the capabilities of the hardware used for the Talisman Saber exercise; however, they do not handle disturbances, such as communication jamming.

## 7. Conclusions

In this paper, a path planning problem for a team of UAVs patrolling a network of roads and pursuing intruders using UGSs is formulated. The problem is shown to be NP-hard. A heuristic algorithm to solve the problem is presented, and its completeness and complexity are assessed. The heuristic primarily plans the paths for the UAVs; however, it also interacts with the UGSs by acquiring information from the UGSs through the UAVs, using the information to predict possible intruder paths and relying on the UGSs to trigger the capture of an image of the intruder by a loitering UAV.

The heuristic demonstrates that intercepting the intruder is possible using a sensing scheme relying entirely on UGSs. Given a good topology for the road network and well selected revisit deadlines, such as Scenario B in Section 5, the heuristic performs well and can intercept a majority of the intruders. The heuristic also exhibits intuitive behavior at times, such as orbiting several UGSs nearby and trapping the intruder, thereby forcing an interception.

In future work, other heuristics to solve the problem and decentralized approaches will be investigated. The ability to handle multiple intruders simultaneously will also be included. This could be achieved by the UGSs tagging their measurements to certain intruders. Variable levels of communication between the UGSs and UAVs will also be considered.

## Figures and Tables

**Table 1.** Base and unattended ground sensor (UGS) parameters for Scenarios A and B.

	**Scenarios A**			**Scenarios B**		

Destination	*ξ_i_*	*ζ_i_*	*r_i_*	*ξ_i_*	*ζ_i_*	*r_i_*
Base	6	6	N/A	6	6	N/A
UGS 1	94.66	70.33	11.4	150.03	56.21	8.1
UGS 2	117.05	109.94	12.4	162.18	124.23	10.7
UGS 3	57.17	151.44	10.5	37.41	72.06	3.0
UGS 4	70.00	100.00	2.6	117.54	131.08	8.4
UGS 5	10.79	106.16	10.8	19.65	110.68	9.5
UGS 6	186.80	25.98	8.3	44.29	4.66	7.1
UGS 7	93.88	2.38	5.6	148.70	107.66	12.6
UGS 8	40.00	50.00	7.0	N/A	N/A	N/A
UGS 9	140.00	42.00	11.0	N/A	N/A	N/A

**Table 2.** Vehicle parameters for Scenarios A and B.

	**Scenario A2**		**Scenario A3**		**Scenario B1**		**Scenario B2**	

Vehicle	*μ_v_*	*σ_v_*	*μ_v_*	*σ_v_*	*μ_v_*	*σ_v_*	*μ_v_*	*σ_v_*
Intruder	37.5	1.57	25	1.57	25	2.33	25	2.33
UAVs	75	0	75	0	25	0	50	0

## Appendix

### A. Local Search Heuristic

**Algorithm A1**: The localSearch() heuristic to find a local minimal cost action for the UAVs.	

	**Data**: *y*(*k*),*f*(·),*x*(·),*S*,*P*(*S*), *search_init_*, *search_iter_*	
1	*Ũ* ← ∅; *u* ← ∅; *ϕ*← ∞	
2	**for** *l* ← 1 **to** *search_init_* **do**	
3	*κ* ← Generate a feasible sequence of actions of a given depth
4	*S, P*(*S*) ← Compute possible intruder paths for the duration of the sequence of actions
5	*i* ← −1
6	*Ũ* ← Compute the k-neighborhood of the sequence of actions
7	*S*, *P*(*S*) ← Extend possible intruder paths to the maximum end time of the k-neighborhood
8	*Ũ.cost* ← Compute costs for all actions
9	$\kappa_{2}\leftarrow \underset{\tilde{u}\in \tilde{U}}{\min}\left(\tilde{u}.\text{cos}t\right)$
10	**while** *κ*_2_ ≠ *κ* Λ *i* < *search_iter_* **do**
11	*κ* ← *κ*_2_
12	*U˜* ← Compute the k-neighborhood of the sequence of actions
13	*S*, *P*(*S*) ← Extend possible intruder paths to the maximum end time of the k-neighborhood
14	*U˜.cost* ← Compute costs for all actions
15	$\kappa_{2}\leftarrow \underset{\tilde{u}\in \tilde{U}}{\min}\left(\tilde{u}.\text{cos}t\right)$
16	**if** *search_iter_* > 0 Λ *i* < 0 **then**
17	*i* ← *i* + 2
18	**else if** *search_iter_* > 0 **then**
19	*i* ← *i* + 1
20	**if** *κ*_2_*.cost* < *ϕ* **then**
21	*u* ← *κ*_2_
	**Result**: *u*

For comparison, a local search heuristic is also implemented to solve this problem (Algorithm A1). At each step, the algorithm randomly generates a feasible sequence of actions of a given depth (Line 3) and computes possible intruder paths that can occur in the same time window (Line 4). The k-neighborhood of the initial sequence of actions is then computed (Line 6) for k = 2, and the possible intruder paths are extended due to the increased time window (Line 7). For each sequence of actions, the corresponding cost is computed using the intruder paths (Line 8), and the minimum cost sequence of actions is extracted (Line 9). This procedure of generating a k-neighborhood and selecting the optimal sequence is repeated until convergence of the minimal cost sequence of actions or a number of iterations, *search_iter_*, is reached (Lines 10–21). The overall procedure can also be repeated for multiple initial random feasible sequences of actions to further increase the search space. *search_init_* indicates the number of these initial guesses. For the search to continue until convergence, *search_iter_* is indicated as zero. The simulations shown in this paper use *search_init_* = 5 and *search_iter_* = 0.
